# Microaxial pump‐supported coronary surgery without CPB to optimize outcome in severely impaired left ventricles

**DOI:** 10.1002/ehf2.15261

**Published:** 2025-04-24

**Authors:** Anna Kathrin Assmann, Merve Arik‐Doganay, Sebastian Waßenberg, Payam Akhyari, Artur Lichtenberg, Alexander Assmann

**Affiliations:** ^1^ Department of Cardiac Surgery, Medical Faculty Heinrich Heine University Duesseldorf Germany; ^2^ punkt05 Statistics Consultants, Life Science Centre Duesseldorf Germany; ^3^ Department for Thoracic and Cardiovascular Surgery University Hospital Essen Essen Germany

**Keywords:** Off‐pump coronary artery bypass grafting, Microaxial pump (Impella 5), Mechanical circulatory support, Ischaemic cardiomyopathy, Severely impaired left ventricular function

## Abstract

**Aims:**

Cardiopulmonary bypass (CPB) is the standard approach for coronary artery bypass grafting (CABG) in advanced ischaemic cardiomyopathy. Microaxial pump support has been envisioned to allow for beating‐heart CABG without CPB (MPCAB), thereby avoiding CPB‐inherent complications. This study aims to compare the in‐hospital and follow‐up outcome of MPCAB versus CPB‐CABG in patients with severely impaired left ventricular function.

**Methods and results:**

Eleven patients suffering from three‐vessel coronary artery disease with median ejection fraction of 27% and deemed appropriate for CABG according to a heart team decision underwent MPCAB (support up to 5.5 L/min). Propensity score matching generated a CPB‐CABG control group (*n* = 33). The primary endpoint was defined as death from any cause by the end of the follow‐up (up to 4 years). MPCAB enabled continuous intraoperative and postoperative haemodynamic stabilization and complete myocardial revascularization. After CPB‐CABG, additional mechanical circulatory support was required in 45.5% (vs. 9.1% in MPCAB; *P* = 0.0363). The follow‐up all‐cause mortality after MPCAB amounted to 0% (vs. 33.3% after CPB‐CABG; *P* = 0.0414; NNT = 3). MPCAB patients showed a significantly decreased occurrence of major adverse cardiovascular events (MACE: 0% vs. 39.4%; *P* = 0.0189).

**Conclusions:**

MPCAB allows for complete surgical revascularization without the necessity of extracorporeal circulation in spite of severely impaired left ventricular function. This first comparative study on the outcome after MPCAB versus CPB‐CABG demonstrates a significantly decreased risk of death as well as MACE in MPCAB patients. The MPCAB concept expands the spectrum of patients eligible for CABG without CPB towards patients with severely impaired left ventricular function.

## Introduction

In coronary artery disease (CAD) patients with severely impaired left ventricular function (LVF), coronary artery bypass grafting (CABG) has been shown to improve survival as compared to optimized medical therapy^1^ and seems to result in superior outcome (mortality, myocardial infarction, and repeat revascularization) when compared to percutaneous coronary intervention.^2^, ^3^, ^4^, ^5^

CABG with haemodynamic support by cardiopulmonary bypass (CPB) is a standard approach to coronary surgery in these patients. Unfortunately, heart–lung machine‐inherent risks predispose to severe intraoperative and postoperative complications, such as arterial wall lesion and atherosclerotic plaque mobilization (due to cannulation, clamping or the pressure washer effect of the aortic cannula jet stream), air embolism, thrombus formation, haemorrhage, and systemic inflammation.

**Figure 1:**
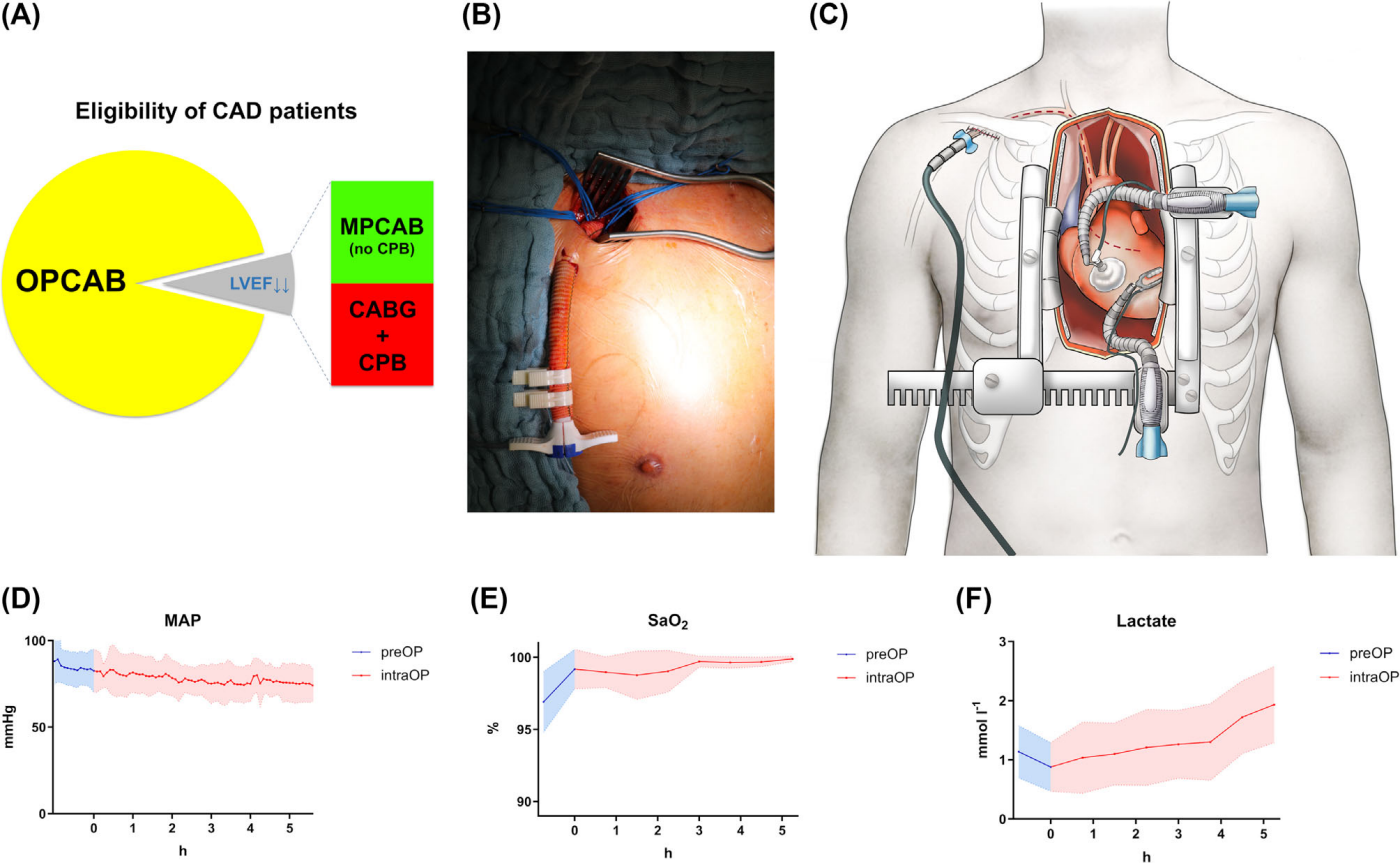
MPCAB technique. (A) MPCAB expands the spectrum of CAD patients eligible for beating‐heart CABG without CPB towards patients with severely impaired LVF. (B) MP sheath in a Dacron graft anastomosed to the right axillary artery. (C) Schematic displaying the MPCAB setting with introduced MP as well as OPCAB positioner and stabilizer. (D–F) Operative monitoring of mean arterial pressure (MAP), arterial oxygen saturation (SaO_2_), and arterial lactate in MPCAB patients.

Off‐pump coronary artery bypass grafting (OPCAB), if performed by an experienced team, allows for decreased mortality as compared to CPB‐CABG in patients with preserved as well as reduced LVF.^6^, ^7^ Minimization of aortic manipulation during OPCAB further reduces operative complications, including a dramatic reduction of stroke risk.^8^, ^9^ Experienced OPCAB teams achieve stable intraoperative haemodynamics in up to 95% of all isolated CABG procedures, including patients with acute coronary syndromes (*Figure* *1* *A*). However, some patients with low cardiac output syndrome (LCOS) due to severe acute ischaemia or chronically severely impaired LVF are not eligible for OPCAB. Unfortunately, exactly, these patients typically present with progressed multimorbidity frequently including huge atherosclerotic plaque burden so that the risk of CPB‐associated complications is high, and these patients should particularly profit from surgery without CPB.

After CPB‐CABG, the combination of surgical trauma, potential intraoperative ischaemia, and the contact of blood with air and the inner surface of the heart–lung machine result in enhanced systemic inflammation. In this situation, patients with severely impaired LVF frequently are unable to reach sufficient cardiac output and thus are prone to develop relative LCOS, due to which postoperative mechanically circulatory support (MCS) is beneficial or even inevitable. Microaxial pump (MP) support with high blood flow rates (up to 5.5 L/min) allows for haemodynamic stabilization of patients with severely impaired LVF^10^, ^11^ and therefore is proposed to enable beating‐heart CABG without CPB (MPCAB) as well as postoperative bridging to recovery in this cohort of high‐risk cases. The feasibility of this concept has been demonstrated in an early pilot series.^12^

The present study aims to compare the in‐hospital and follow‐up outcome of MPCAB versus CPB‐CABG in patients with severely impaired LVF.

## Methods

### Patients and data acquisition

Eleven adult patients with ischaemic cardiomyopathy (left ventricular ejection fraction [LVEF] ≤ 35%) due to three‐vessel disease and deemed appropriate for CABG according to a heart team decision underwent isolated MPCAB at a single institution (2019–2023). Exclusion criteria comprised severe valvular disease, left ventricular aneurysm with surgical indication, severe aortic pathology (aneurysm, dissection, or atherosclerosis ≥ grade IV), severely impaired right ventricular function, acute cardiogenic shock requiring inotropic support, and redo cardiac surgery.

The propensity score‐based control group (*n* = 33) was generated from the cohort of all isolated CPB‐CABG patients with LVEF ≤ 35% in the same time period (*n* = 71). All in‐hospital parameters were prospectively recorded and collected from the data acquisition system of the hospital. Post‐discharge follow‐up data at 32 [IQR, 17–44] months were assessed by structured phone calls in 97.7% of all patients.

The study was approved by the local ethics committee (09/18/2023; approval number 2023‐2472) and complies with the Declaration of Helsinki. Patient care followed the standardized hospital guidelines. Patients' informed written consent statement was obtained.

### Surgical technique

MP implantation (Impella 5.0® or Impella 5.5®, Abiomed, Aachen, Germany) was the first step of all MPCAB operations to allow for early intraoperative haemodynamic stabilization. Preoperatively, computed tomography scanning of the chest had been performed to rule out the presence of severe stenotic or mobile plaques in the vasculature between the right axillary artery and the aortic valve and vascular diameters or malformations that jeopardize the safe insertion of an MP catheter. Intraoperatively, after exposition and preparation of the right axillary artery, a gelatin‐sealed Dacron vascular graft (d = 10 mm) was anastomosed in an end‐to‐side manner (*Figure* *1* *B*). Following moderate heparinization (target activated clotting time 200–250 s), an MP catheter was inserted and positioned by means of the Seldinger technique under direct visualization by radiography as well as transesophageal echocardiography according to the manufacturer's recommendation (*Video* *1*). MP support (up to 5.5 L/min) was installed, and MPCAB was conducted by an experienced OPCAB team (including specialized surgeons as well as cardioanesthetists) following our standardized approach as previously published.^8^, ^12^, ^13^ In brief, automated ST segment analysis and central venous pressure as well as pulmonary artery pressure readings and continuous cardiac output measurements were applied for haemodynamic monitoring. Internal thoracic arteries were harvested in a skeletonized manner. Pericardial slings, apical suction positioner devices, blower/mister devices, intracoronary shunts, and bypass flow measurements were routinely used. In the case of aortic manipulation, epiaortic ultrasound and clampless anastomotic devices were utilized throughout. *Figure* *1* *C* displays a schematic of the MPCAB setting.

**VIDEO 1:** MP catheter insertion and positioning control. Dual imaging (radiography and transesophageal echocardiography) guides the MP implantation process.

The CPB‐CABG approach contained multimodal blood cardioplegia (antegrade, retrograde via the coronary sinus, and via coronary bypasses) and aortic cross‐clamping as well as side‐clamping for proximal anastomoses.

### Endpoints

The primary endpoint was defined as death from any cause over the whole study period. Secondary endpoints comprised the occurrence of MACE (all‐cause death, myocardial infarction according to the fourth universal definition of myocardial infarction,^14^ repeat revascularization) and MACCE (MACE + stroke according to the ‘Updated definition of stroke for the 21st century: a statement for healthcare professionals from the American Heart Association/American Stroke Association’^15^) by the end of the follow‐up, as well as further in‐hospital and follow‐up outcome parameters.

### Statistics

Propensity score matching was conducted by means of the optimal matching algorithm in order to minimize the average difference across all matched pairs.^16^ The preoperative matching variables comprised age, sex, body mass index (BMI), Canadian Cardiovascular Society (CCS) class, New York Heart Association (NYHA), American Society of Anesthesiologists (ASA) class, acute myocardial infarction < 48 h, cardiopulmonary reanimation (CPR) < 48 h, atrial fibrillation (AF), LVEF, arterial hypertension (HTN), diabetes mellitus (DM‐II), hyperlipidaemia, troponin‐Ths level, and emergency operation.

Descriptive statistics are presented as median and interquartile range (IQR) for all quantitative variables. Following normality testing (D'Agostino–Pearson test), direct group comparisons were conducted by two‐tailed Mann–Whitney *U* test or Student's *t*‐test, respectively. Regarding categorical variables, pairwise Fisher's exact or chi‐square tests were performed. For the main endpoint variables, the effect size was measured using Cramer's V (≤0.2 weak; >0.2 and ≤0.4 moderate; >0.4 strong). In order to evaluate survival and freedom from major adverse events, Kaplan–Meier estimates, allowing for censored data, were applied and analysed by log‐rank (Mantel–Cox) tests.

Significance was assumed for *P* values <0.05. Data analysis was conducted with SPSS Statistics for Windows (IBM, Armonk, NY, USA) and GraphPad Prism v8.02 (GraphPad Software, San Diego, USA).

## Results

### Propensity score matching

Thirty‐three CPB‐CABG patients were assigned to the 11 MPCAB cases. The overall balance test showed a clear balance of all covariates (*P* = 0.9843).^17^ The matching quality was further confirmed by receiver operating characteristic curve analysis (area under the curve 0.88 [95% CI 0.80–0.97, *P* < 0.0001]). After propensity score matching, preoperative baseline characteristics did not significantly differ between MPCAB and CPB‐CABG patients (*Table* *1*).

**Table 1:** Baseline characteristics of patients undergoing MPCAB versus CPB‐CABG after propensity score matching

Variable	MPCAB (*n* = 11)	CPB‐CABG (*n* = 33)	*P* value
Age (y)	63 [58–71]	66 [59–75]	0.3257
Sex male	100	100	>0.9999
BMI (kg m^−2^)	26 [24–27]	25 [23–29]	0.5902
CPR < 48 h	0.0	3.0	>0.9999
LVEF (%)	27 [25–35]	29 [25–30]	0.5236
CCS ≥ 3	63.6	72.7	0.7058
NYHA ≥ 3	72.7	81.8	0.6686
AF	18.2	15.1	>0.9999
Stroke	9.1	12.1	>0.9999
CAD	45.5	15.2	0.0899
PVD	9.1	21.2	0.6563
HTN	72.7	81.8	0.6686
COPD	18.2	15.2	>0.9999
DM II	36.4	30.3	0.7222
Troponin‐Ths (ng L^−1^)	56 [24–751]	110 [36–624]	0.6506
Creatinine (mg dL^−1^)	1.0 [0.9–1.1]	1.0 [0.9–1.3]	0.9408
GFR (mL min^−1^)	89 [69–90]	77 [57–93]	0.5605
Elective surgery	54.6	36.4	0.3141
EuroScore II (%)	2.5 [2.0–7.0]	5.5 [2.5–7.7]	0.4566
SYNTAX score	30 [18–47]	27 [19–33]	0.2792

*Note*: Quantitative variables are displayed as median [IQR], and categorical variables as percentage.

AF, atrial fibrillation; BMI, body mass index; CAD, carotid artery disease; CCS, Canadian Cardiovascular Society classification; COPD, chronic obstructive pulmonary disease; CPB‐CABG, coronary artery bypass grafting with cardiopulmonary bypass; CPR, cardiopulmonary resuscitation; DM II, diabetes mellitus type II; GFR, glomerular filtration rate; HTN, arterial hypertension; LVEF, left ventricular ejection fraction; MPCAB, microaxial pump‐supported beating‐heart CABG without CPB; NYHA, New York Heart Association classification; PVD, peripheral vascular disease.

### Intraoperative parameters

MPCAB patients showed stable haemodynamics (*Figure* *1* *D*–*F*) without conversion to CPB and complete revascularization (*Table* *2*).

**Table 2:** Intraoperative variables of patients undergoing MPCAB versus CPB‐CABG

Variable	MPCAB (*n* = 11)	CPB‐CABG (n = 33)	*P* value
Cut‐suture time (min)	351 [309–379]	225 [143–299]	**<0.0001**
Graft number (*n*)	2 [2–3]	3 [2–3]	0.3951
Peripheral anastomoses (*n*)	3 [2–4]	3 [2–3]	0.7408
Peripheral anastomoses with arterial grafts (*n*)	1 [1–1]	1 [1–1]	0.7072

*Note*: Quantitative variables are displayed as median [IQR].

CPB‐CABG, coronary artery bypass grafting with cardiopulmonary bypass; MPCAB, microaxial pump‐supported beating‐heart CABG without CPB.

### In‐hospital outcome

Preoperatively, none of the patients required MCS. In the CPB‐CABG group, 45.5% of the patients had to undergo MCS implantation after CPB (extracorporeal life support [ECLS], MP, or intra‐aortic balloon pump [IABP]) versus 9.1% after MPCAB (*P* = 0.0363; Cramer's V 0.33). Twelve out of 15 MCS implantations were conducted directly at the end of the operation due to insufficient weaning from CPB, while three patients received MCS on the intensive care unit (ICU) at postoperative day 1 or 2. The necessity of ECLS after CPB‐CABG amounted to 24.2% versus 9.1% after MPCAB (*P* = 0.4108), with a median ECLS duration of 8 [IQR, 5–11] days. Among eight patients with ECLS after CPB‐CABG, four died on ECLS, two were bridged to left ventricular assist device implantation, and only two could be weaned from ECLS, whereas one of the latter two patients died 2 weeks after transfer to another hospital. On the contrary, weaning from MCS was successful in 90.9% of MPCAB patients (while one patient was bridged to left ventricular assist device implantation), with a median MP support duration of 5 [IQR, 4–10] days. The one MPCAB patient requiring ECLS on postoperative day 2, due to MP dislocation in the context of patient transfer for redo thoracotomy, could be successfully weaned from ECLS 4 days later. Further in‐hospital outcome is displayed in *Table* *3*.

**Table 3:** In‐hospital outcome of patients undergoing MPCAB versus CPB‐CABG

Variable	MPCAB (*n* = 11)	CPB‐CABG (*n* = 33)	*P* value
MCS	9.1	45.5	**0.0363**
ECLS	9.1	24.2	0.4108
*Only 1 out of 8 CPB‐CABG patients on ECLS was weaned + discharged at home after MPCAB; 1 patient required ECLS due to MP dislocation*			
Redo thoracotomy (bleeding)	27.3	15.6	0.4010
Creatinine (mg dL^−1^)	1.2 [1.1–1.4]	1.3 [1.0–1.8]	0.5328
Renal replacement therapy	0	9.1	0.5615
CK‐MB_max_ (U L^−1^)	37 [20–42]	54 [34–78]	**0.0048**
Troponin‐Ths_max_ (ng L^−1^)	725 [510–1132]	1,391 [670–2511]	0.1292
Troponin‐Ths_post‐preop_ (ng L^−1^)	589 [396–1002]	997 [452–1808]	0.0870
LVEF_post‐preop_ (%)	5 [0–9]	0 [0–2]	**0.0357**
ICU stay duration (h)	163 [114–412]	79 [24–189]	**0.0251**
Hospital stay duration (d)	25 [14–30]	10 [8–14]	**0.0007**
Discharge at home	63.6	42.4	0.3028
In‐hospital stroke	9.1	21.2	0.6563
In‐hospital mortality	0	21.2	0.1652
*Cramer's V effect size 0.25*			

*Note*: Quantitative variables are displayed as median [IQR], and categorical variables as percentage. Values in bold indicate statistical relevance.

CK‐MB, creatine kinase isoenzyme MB; CPB‐CABG, coronary artery bypass grafting with cardiopulmonary bypass; ECLS, extracorporeal life support; ICU, intensive care unit; LVEF, left ventricular ejection fraction; MCS, mechanical circulatory support; MPCAB, microaxial pump‐supported beating‐heart CABG without CPB.

### Primary endpoint: Mortality (follow‐up)

**Figure 2:**
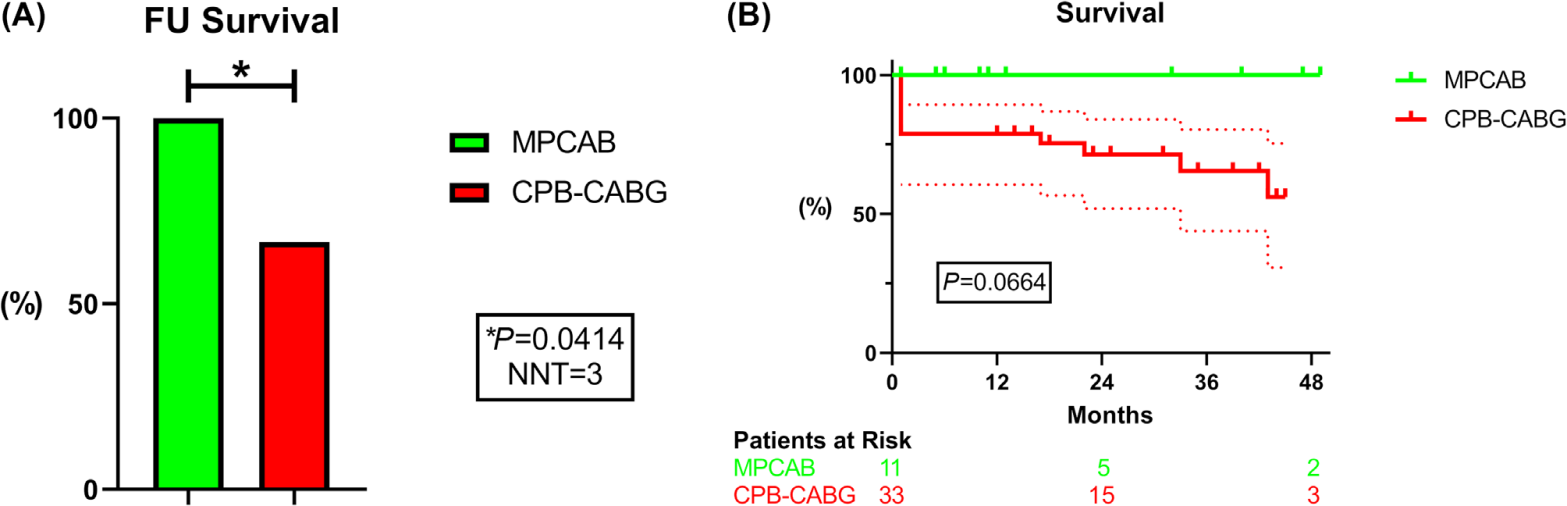
Survival after MPCAB versus CPB‐CABG. MPCAB patients showed remarkably improved survival (A) at the end of the FU and (B) over the whole study period. NNT, number needed to treat.

The all‐cause mortality over the whole study period was significantly decreased in the MPCAB group as compared to CPB‐CABG (0% vs. 33.3%, *P* = 0.0414; Cramer's V 0.33), with a number needed to treat (NNT) of 3 (95% CI 1.46–6.78) (*Figure* *2* *A*). Based on a Kaplan–Meier estimate, the hazard ratio (HR) in the CPB‐CABG group was 4.22 (95% CI 0.95–18.7, *P* = 0.0664) (*Figure* *2* *B*).

### Secondary follow‐up endpoints

**Figure 3:**
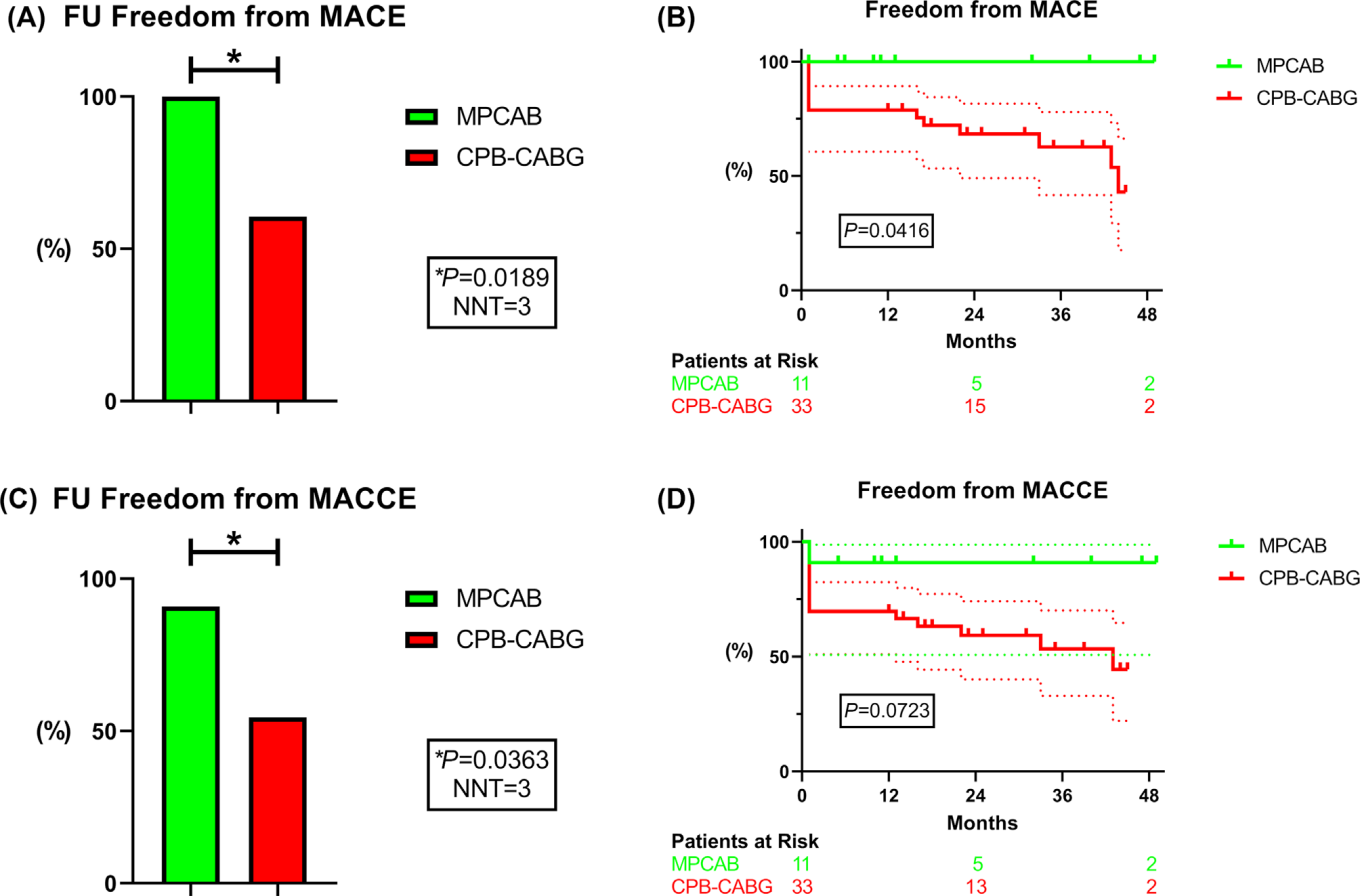
MACE and MACCE after MPCAB versus CPB‐CABG. MPCAB patients showed remarkably improved MACE/MACCE (A,C) at the end of the FU and (B,D) over the whole study period. NNT, number needed to treat.

The occurrence of MACE by the end of the follow‐up was significantly increased in the CPB‐CABG group (39.4% vs. 0% in MPCAB, *P* = 0.0189; Cramer's V 0.37; Kaplan–Meier‐based HR 4.14 with 95% CI 1.06–16.2, *P* = 0.0416) (*Figure* *3* *A,B*). The MACCE rate in CPB‐CABG patients was significantly increased as well (45.5% vs. 9.1% in MPCAB, *P* = 0.0363; Cramer's V 0.33), while the respective Kaplan–Meier‐based HR amounted to 3.19 (95% CI 0.90–11.3, *P* = 0.0723) (*Figure* *3* *C,D*).

The higher stroke rate in CPB‐CABG patients at the end of the follow‐up did not reach statistical significance (27.3% vs. 9.1%, *P* = 0.4082). Only one MPCAB patient with ECLS on day 2 developed a posterior inferior cerebellar artery infarction and survived the follow‐up. On the contrary, in CPB‐CABG patients, nine strokes occurred, out of which seven were diagnosed following postoperative MCS, and five affected patients who died during the follow‐up (four of these five patients died only a few days after stroke).

At the end of the follow‐up, 78% of MPCAB patients were free from dyspnoea (NYHA class I; vs. 45% in the CPB‐CABG group), while only CPB‐CABG patients suffered from NYHA class ≥III (25% vs. 0% in the MPCAB group; *P* = 0.1657 across all NYHA classes).

## Discussion

The present study is—to the best of our knowledge—the first report on comparative evaluation of the outcome after MPCAB versus CPB‐CABG in patients with severely impaired LVF. We demonstrate that our MPCAB approach decreases mortality as well as MACE and MACCE. Additionally, we prove the safety and feasibility of this technique, as well as the potential of MPCAB to enable gentle postoperative haemodynamic weaning without CPB in this cohort of high‐risk patients.

### Intraoperative haemodynamic support

Recently, we reported on our early experience with MPCAB to allow for beating‐heart CABG without CPB in three patients with severely impaired LVF.^12^ Now, we demonstrate the safety of this approach, as well as the feasibility in terms of adequate intraoperative haemodynamic support, in a cohort of consecutive patients.

After MP positioning, stable haemodynamics were achieved and maintained in all patients, which is of utmost importance, since even short periods of insufficient left ventricular output may cause pulmonary vascular congestion that rapidly diminishes pulmonary gas exchange triggering a vicious circle. As a consequence, low arterial lactate levels were measured indicating aerobic metabolism due to adequate organ perfusion. MP support enabled cardiac luxation for bypass anastomoses in basal areas of the lateral and posterior wall and thus complete revascularization without CPB in all patients.

### Postoperative haemodynamic support and weaning

Relative LCOS is a typical issue after CABG in patients with low LVEF, which predominantly results from systemic inflammation due to surgical trauma and MCS‐inherent inflammatory activation.^18^, ^19^ Particularly after CPB‐CABG, relative LCOS can be even aggravated by intraoperative ischaemia that not rarely occurs under cardioplegic arrest of hearts with progressed CAD.

In the case of relative LCOS after CPB‐CABG, common strategies comprise early MCS (ECLS or MP) initiation or a conservative attempt with high risk of consecutive emergency ECLS implantation in cardiogenic shock.^20^, ^21^ Patients who require ECLS after CABG exhibit unfavourable outcome, such as in‐hospital mortality far higher than 50%.^22^, ^23^, ^24^, ^25^ In the present study, the rate of postoperative MCS implantations due to relative LCOS after CPB‐CABG was high, and the outcome among those who required ECLS was alarming, with five out of eight ECLS patients dying early and only one out of eight patients surviving after myocardial recovery. On the contrary, MPCAB patients presented with stable postoperative haemodynamics. Beyond the choice of MCS modality, the aspect of timing in terms of early support for patients at high risk of postoperative haemodynamical instability has to be considered.

MP therapy does not only guarantee systemic organ perfusion but additionally protects the myocardium from left ventricular volume overload, which frequently occurs under sole ECLS in patients with severely impaired LVF as a consequence of enhanced left ventricular afterload due to the ECLS‐generated continuous aortic pressure. Therefore, left ventricular unloading has been implemented in state‐of‐the‐art strategies of cardiac failure treatment and is recommended in current ECLS guidelines.^26^, ^27^, ^28^

Volume unloading reduces left ventricular wall shear stress and intramural pressure thereby improving myocardial perfusion so that additional postoperative myocardial damage is prevented, and a timeframe for myocardial recovery is generated.^29^ The superior postoperative development of the LVEF and the postoperative serum markers of myocardial injury suggest an advantage of MPCAB over CPB‐CABG in terms of myocardial recovery. As recovery progresses, stepwise reduction of left ventricular unloading allows for gentle weaning from MCS. In the present study, all MPCAB patients were successfully weaned from MP support except one patient who required left ventricular assist device therapy.

Summarizing this subchapter, MPCAB allows for postoperative haemodynamic stabilization with adequate systemic perfusion and maintained blood flow in the pulmonary vasculature and the cardiac cavities, as well as left ventricular unloading and gentle weaning from MCS, which seem to facilitate myocardial recovery. In opposite to CPB‐CABG approaches, only one MCS device is required, which—besides all medical advantages—additionally has economic implications.

### Follow‐up outcome

Analysis of the primary endpoint revealed a significantly decreased mortality in the MPCAB group as compared to CPB‐CABG after up to 4 years (NNT = 3 patients only). The majority of deaths in the CPB‐CABG group occurred as a consequence of perioperative stroke or failed weaning from MCS. Both scenarios are typical complications under heart–lung machine support (CPB‐CABG and/or ECLS) in patients with low LVF and advanced atherosclerosis. The mortality rate in our CPB‐CABG group is alarmingly high, whereas the data are in line with previous publications on mortality in patients requiring MCS after isolated CABG.^25^, ^30^ Nevertheless, the high mortality rate actually was a driving force to develop an alternative, less invasive operative approach.

Driven by the remarkable survival advantage, the risk of MACE and MACCE was also significantly decreased in the MPCAB group as compared to CPB‐CABG. Due to a single stroke, the lower follow‐up stroke rate in MPCAB patients did not reach statistical significance. However, it occurred after emergency ECLS implantation due to MP dislocation during patient transfer to the operation room. On the one hand, this event underlines the importance of careful MP handling, but on the other side, it also points at the risk of adverse events during ECLS and emergency MCS in particular. Furthermore, it has to be noticed that the stroke event in the MPCAB group was a small posterior inferior cerebellar artery infarction, and the patient survived the follow‐up without major neurological limitations. On the contrary, the majority of the nine stroke patients in the CPB‐CABG group died shortly after stroke.

### Microaxial pump pitfalls

Our MPCAB approach exhibits logical technical advantages as well as improvement of clinical outcome. Nevertheless, identification of patients who may not profit from MP support is essential. Preoperative routine should include a chest computed tomography scan to rule out the presence of severe stenotic or mobile plaques in the vasculature between the right axillary artery and the aortic valve and vascular diameters or malformations that jeopardize the safe insertion of an MP catheter. Furthermore, sole MP support may not suffice for patients with additional pulmonary and/or right ventricular failure.

Based on our experience, usage of MP models with lower maximum pump flow is not favourable to MPCAB, as adequate exposition of complex bypass targets on dilated left ventricles not rarely necessitates more intense luxation. In these scenarios, high pump flows (up to 5.5 L/min) accomplish sufficient systemic perfusion. Postoperatively, high pump flows avoid insufficient cardiac unloading and relative LCOS. This statement in favour of ‘large’ MP systems is strongly influenced by our experience with MP support in cardiac failure patients with or without additional ECLS.^31^, ^32^

Intraoperative positioning of the MP catheter visually guided by radiography and transesophageal echocardiography, as well as careful fixation and intermittent positioning control are strongly recommended to avoid accidental dislocation—particularly during cardiac luxation and patient transfer—that may have fatal consequences.

These data show a relevant, albeit not significantly enhanced, rate of redo thoracotomies due to bleeding after MPCAB. This observation requires further evaluation in future studies and may be beneficially influenced by the choice of the MP purge fluid as well as haemotherapeutic approaches.

In the present study, the ICU and in‐hospital stay were significantly longer after MPCAB versus CPB‐CABG. In the face of superior outcome after MPCAB, this aspect may appear subordinate; nevertheless, the economic effects on the healthcare system should be addressed by future research.

Considering all aforementioned challenges, adoption of the MPCAB concept for the treatment of high‐risk patients with severely impaired left ventricles is recommended to be preceded by profound experience in OPCAB techniques, concerning the surgical as well as the anaesthesiological team members, and expertise in MP handling that can be gained in heart failure programmes.

### Limitations

The observational retrospective study design is considered as limitation, so potential selection bias has been balanced by propensity score matching of the control cohort. The small sample size is also a limitation of the present study, whereas CABG in severely impaired LVF is not a highly frequent event, so large cohorts cannot be expected. Moreover, many of these patients are eligible to undergo unprotected OPCAB without MCS and thereby further reduce the number of patients that fit the purpose of the present study.

Nevertheless, even with this low sample number, the outcome of MPCAB patients was significantly improved, which was corroborated by effect size measures that yielded moderate effects for all respective variables. In our study, MPCAB has been compared to CPB‐CABG including aortic cross‐clamping and cardioplegia. It may be of interest to additionally compare MPCAB to beating‐heart CPB‐CABG; however, the aim of this examination was to evaluate the outcome of MPCAB versus our standard technique for CABG in patients with severely impaired LVEF.

## Conclusions

In this first comparative study on the outcome after MPCAB versus CPB‐CABG in patients with severely impaired LVF, we demonstrate decreased mortality as well as MACE and MACCE in the MPCAB group, whereas the small patient numbers of our study have to be considered. By means of MP support, intraoperative and postoperative haemodynamic stabilization, and postoperatively adequate systemic perfusion, prevention of pulmonary vascular or cardiac blood stasis, left ventricular unloading and gentle weaning from MCS can be achieved, which seems to facilitate myocardial recovery. The MPCAB concept expands the spectrum of patients who are eligible to undergo beating‐heart CABG without CPB towards patients with severely impaired LVF. Particularly patients with high risk of CPB‐associated complications, such as patients with extensive non‐coronary atherosclerosis, may profit from MPCAB.

## Funding

None.

## Conflict of interest

P.A. is PI of IMpella‐Protected cArdiaC Surgery Trial and consultant for Abiomed and Medtronic.

## References

[1] Velazquez EJ , Lee KL , Jones RH , Al‐Khalidi HR , Hill JA , Panza JA , *et al*. Coronary‐artery bypass surgery in patients with ischemic cardiomyopathy. N Engl J Med 2016;374:1511‐1520. doi:10.1056/NEJMoa1602001 27040723 PMC4938005

[2] Wolff G , Dimitroulis D , Andreotti F , Kołodziejczak M , Jung C , Scicchitano P , *et al*. Survival benefits of invasive versus conservative strategies in heart failure in patients with reduced ejection fraction and coronary artery disease: a meta‐analysis. Circ Heart Fail 2017;10:e003255. doi:10.1161/CIRCHEARTFAILURE.116.003255 28087687

[3] Gaudino M , Hameed I , Khan FM , Tam DY , Rahouma M , Yongle R , *et al*. Treatment strategies in ischaemic left ventricular dysfunction: a network meta‐analysis. Eur J Cardiothorac Surg 2020;59:293‐301. doi:10.1093/ejcts/ezaa319 33085752

[4] Nagendran J , Bozso SJ , Norris CM , McAlister FA , Appoo JJ , Moon MC , *et al*. Coronary artery bypass surgery improves outcomes in patients with diabetes and left ventricular dysfunction. J Am Coll Cardiol 2018;71:819‐827. doi:10.1016/j.jacc.2017.12.024 29471931

[5] Sun LY , Gaudino M , Chen RJ , Bader Eddeen A , Ruel M . Long‐term outcomes in patients with severely reduced left ventricular ejection fraction undergoing percutaneous coronary intervention vs coronary artery bypass grafting. JAMA Cardiol 2020;5:631‐641. doi:10.1001/jamacardio.2020.0239 32267465 PMC7142806

[6] Benedetto U , Lau C , Caputo M , Kim L , Feldman DN , Ohmes LB , *et al*. Comparison of outcomes for off‐pump versus on‐pump coronary artery bypass grafting in low‐volume and high‐volume centers and by low‐volume and high‐volume surgeons. Am J Cardiol 2018;121:552‐557. doi:10.1016/j.amjcard.2017.11.035 29291888

[7] Keeling WB , Williams ML , Slaughter MS , Zhao Y , Puskas JD . Off‐pump and on‐pump coronary revascularization in patients with low ejection fraction: a report from the Society of Thoracic Surgeons national database. Ann Thorac Surg 2013;96:83‐88. doi:10.1016/j.athoracsur.2013.03.098 23743061

[8] Albert A , Ennker J , Hegazy Y , Ullrich S , Petrov G , Akhyari P , *et al*. Implementation of the aortic no‐touch technique to reduce stroke after off‐pump coronary surgery. J Thorac Cardiovasc Surg 2018;156:544‐554.e4. doi:10.1016/j.jtcvs.2018.02.111 29778336

[9] Zhao DF , Edelman JJ , Seco M , Bannon PG , Wilson MK , Byrom MJ , *et al*. Coronary artery bypass grafting with and without manipulation of the ascending aorta. J Am Coll Cardiol 2017;69:924‐936. doi:10.1016/j.jacc.2016.11.071 28231944

[10] Sugimura Y , Bauer S , Immohr MB , Mehdiani A , Rellecke P , Tudorache I , *et al*. Clinical outcomes of hundred large Impella implantations in cardiogenic shock patients based on individual clinical scenarios. Artif Organs 2023;47:1874‐1884. doi:10.1111/aor.14646 37724611

[11] Bossi E , Marini C , Gaetti G , Diamanti L , Alessio D , Bertoldi LF , *et al*. Efficacy and safety of Impella 5.0 in cardiogenic shock: an updated systematic review. Future Cardiol 2022;18:253‐264. doi:10.2217/fca-2021-0046 34713720

[12] Katahira S , Sugimura Y , Mehdiani A , Assmann A , Rellecke P , Tudorache I , *et al*. Coronary artery bypass grafting under sole Impella 5.0 support for patients with severely depressed left ventricular function. J Artif Organs 2022;25:158‐162. doi:10.1007/s10047-021-01285-1 34169403 PMC9142466

[13] Albert A , Assmann A , Assmann AK , Aubin H , Lichtenberg A . Operative Techniques in Coronary Artery Bypass Surgery: An Illustrated Guide to Personalized Therapy. Springer Nature; 2020.

[14] Thygesen K , Alpert JS , Jaffe AS , Chaitman BR , Bax JJ , Morrow DA , *et al*. Fourth universal definition of myocardial infarction (2018). J Am Coll Cardiol 2018;72:2231‐2264. doi:10.1016/j.jacc.2018.08.1038 30153967

[15] Sacco RL , Kasner SE , Broderick JP , Caplan LR , Connors JJ(B) , Culebras A , *et al*. An updated definition of stroke for the 21st century. Stroke 2013;44:2064‐2089. doi:10.1161/STR.0b013e318296aeca 23652265 PMC11078537

[16] Stuart EA . Matching methods for causal inference: a review and a look forward. Stat Sci 2010;25:1‐21. doi:10.1214/09-STS313 20871802 PMC2943670

[17] Hansen BB , Bowers J . Covariate balance in simple, stratified and clustered comparative studies. Stat Sci 2008;23:219‐236. doi:10.1214/08-STS254

[18] Maranta F , Cianfanelli L , Grippo R , Alfieri O , Cianflone D , Imazio M . Post‐pericardiotomy syndrome: insights into neglected postoperative issues. Eur J Cardiothorac Surg 2022;61:505‐514. doi:10.1093/ejcts/ezab449 34672331

[19] Hatami S , Hefler J , Freed DH . Inflammation and oxidative stress in the context of extracorporeal cardiac and pulmonary support. Front Immunol 2022;13:831930. doi:10.3389/fimmu.2022.831930 35309362 PMC8931031

[20] Park SJ , Kim JB , Jung SH , Choo SJ , Chung CH , Lee JW . Outcomes of extracorporeal life support for low cardiac output syndrome after major cardiac surgery. J Thorac Cardiovasc Surg 2014;147:283‐289. doi:10.1016/j.jtcvs.2012.11.006 23219332

[21] Wu MY , Lin PJ , Lee MY , Tsai FC , Chu JJ , Chang YS , *et al*. Using extracorporeal life support to resuscitate adult postcardiotomy cardiogenic shock: treatment strategies and predictors of short‐term and midterm survival. Resuscitation 2010;81:1111‐1116. doi:10.1016/j.resuscitation.2010.04.031 20627521

[22] Mariani S , Heuts S , van Bussel BCT , di Mauro M , Wiedemann D , Saeed D , *et al*. Patient and management variables associated with survival after postcardiotomy extracorporeal membrane oxygenation in adults: the PELS‐1 multicenter cohort study. J Am Heart Assoc 2023;12:e029609. doi:10.1161/JAHA.123.029609 37421269 PMC10382118

[23] Kowalewski M , Zielinski K , Brodie D , MacLaren G , Whitman G , Raffa GM , *et al*. Venoarterial extracorporeal membrane oxygenation for postcardiotomy shock‐analysis of the extracorporeal life support organization registry. Crit Care Med 2021;49:1107‐1117. doi:10.1097/CCM.0000000000004922 33729722 PMC8217275

[24] Rastan AJ , Dege A , Mohr M , Doll N , Falk V , Walther T , *et al*. Early and late outcomes of 517 consecutive adult patients treated with extracorporeal membrane oxygenation for refractory postcardiotomy cardiogenic shock. J Thorac Cardiovasc Surg 2010;139:302‐311.e1. doi:10.1016/j.jtcvs.2009.10.043 20106393

[25] Biancari F , Dalen M , Perrotti A , Fiore A , Reichart D , Khodabandeh S , *et al*. Venoarterial extracorporeal membrane oxygenation after coronary artery bypass grafting: results of a multicenter study. Int J Cardiol 2017;241:109‐114. doi:10.1016/j.ijcard.2017.03.120 28389122

[26] Assmann A , Beckmann A , Schmid C , Werdan K , Michels G , Miera O , *et al*. Use of extracorporeal circulation (ECLS/ECMO) for cardiac and circulatory failure ‐a clinical practice guideline level 3. ESC Heart Fail 2022;9:506‐518. doi:10.1002/ehf2.13718 34811959 PMC8788014

[27] Tongers J , Sieweke JT , Kuhn C , Napp LC , Flierl U , Röntgen P , *et al*. Early escalation of mechanical circulatory support stabilizes and potentially rescues patients in refractory cardiogenic shock. Circ Heart Fail 2020;13:e005853. doi:10.1161/CIRCHEARTFAILURE.118.005853 32164431

[28] Pappalardo F , Schulte C , Pieri M , Schrage B , Contri R , Soeffker G , *et al*. Concomitant implantation of Impella® on top of veno‐arterial extracorporeal membrane oxygenation may improve survival of patients with cardiogenic shock. Eur J Heart Fail 2016;19:404‐412. doi:10.1002/ejhf.668 27709750

[29] Lusebrink E , Binzenhofer L , Kellnar A , Müller C , Scherer C , Schrage B , *et al*. Venting during venoarterial extracorporeal membrane oxygenation. Clin Res Cardiol 2023;112:464‐505. doi:10.1007/s00392-022-02069-0 35986750 PMC10050067

[30] Singh SK , Vinogradsky A , Kirschner M , Sun J , Wang C , Kurlansky P , *et al*. Mechanical circulatory support during surgical revascularization for ischemic cardiomyopathy. Ann Thorac Surg 2024;117:932‐939. doi:10.1016/j.athoracsur.2024.01.017 38302051

[31] Sugimura Y , Katahira S , Immohr MB , Sipahi NF , Mehdiani A , Assmann A , *et al*. Initial experience covering 50 consecutive cases of large Impella implantation at a single heart Centre. ESC Heart Fail 2021;8:5168‐5177. doi:10.1002/ehf2.13594 34480419 PMC8712922

[32] Sugimura Y , Immohr MB , Mehdiani A , Boeken U , Aubin H , Lichtenberg A , *et al*. Impact of Impella support on clinical outcomes in patients with postcardiotomy cardiogenic shock. Ann Thorac Cardiovasc Surg 2024;30: doi:10.5761/atcs.oa.23-00076 PMC10902663 37532525

